# Effective Treatment of Diabetes Mellitus by Resonance Medicine

**DOI:** 10.7759/cureus.29535

**Published:** 2022-09-24

**Authors:** Shyam Jalan, Ashish Anjankar, Shubham Deshpande

**Affiliations:** 1 Anatomy, Jawaharlal Nehru Medical College, Datta Meghe Institute of Medical Sciences, Wardha, IND; 2 Biochemistry, Jawaharlal Nehru Medical College, Datta Meghe Institute of Medical Sciences, Wardha, IND; 3 Physiology, Jawaharlal Nehru Medical College, Datta Meghe Institute of Medical Sciences, Wardha, IND

**Keywords:** nosode-homeopathic treatment, bio-resonance, beta cells, t lymphocytes, transplantation, organo preparation

## Abstract

The metabolic disorder known as diabetes mellitus (DM) has several different causes, distinguished by recurring hyperglycemia due to inadequate insulin secretion, insulin action, or both. T-lymphocytes target such cells for destruction, which include beta cells. Transplants of the pancreas, islets of Langerhans, and individual beta cells are all effective treatments for DM. Additionally, treating DM using stem cells is popular currently. The basis of stem cell therapy for DM is the replacement of beta cells, or dead pancreatic cells, with stem cells. After attaching to the tissues of the pancreas, the stem cells differentiate into active cells. An X-ray scanner is used to place a catheter into the pancreatic artery in DM, and the process lasts 90 minutes. The use of stem cells to replace dead pancreatic beta cells forms the cornerstone of stem cell treatment for DM. Transplants of the pancreas, islets of Langerhans, and individual beta cells are all effective treatments for insulin-dependent DM.

In contrast to prior studies, where we only used low potencies of nosodes and organopreparations, our research used both high and low potencies of these substances. Choosing the strength of the nosode stomach cancer in the computer-connected device selector so that it will resonate with the nosode that is tested in the patient's device is the doctor's responsibility when using the bioresonance therapy method. The initial nosode, which is in the computer programme of the device for bioresonance therapy, is no longer tested when the stomach cancer nosode is tested in a patient along with the chosen potency of this nosode. The initial nosode in the bioresonance therapy device itself is still being studied in case the chosen nosode's potency is inadequate (the frequency of oscillations of the nosode is lower than the frequency of oscillations of the tumour).

## Introduction and background

The metabolic disorder known as diabetes mellitus (DM) has several different causes, distinguish by recurring hyperglycemia developing from inadequate insulin secretion, insulin action, or both. DM is a chronic disease which is an example of a prevalent disease spread in nearly all nations across the world and constitutes a severe public health concern that is even under pandemic scrutiny [1]. The central and/or peripheral neurological systems may be affected by the metabolic problems present in DM, which are frequently characterized by an increase in blood glucose concentration [2]. Long-term harm, dysfunction, and failure of numerous organs, including the kidneys, heart, blood vessels, brain, and nerves, are associated with DM's persistent hyperglycemia. The main reasons for inadequate insulin action include insufficient insulin secretion and/or diminished tissue responsiveness to insulin at one or more sites in the hormonal action chain [3]. Type 1 DM is insulin-dependent. The entire range of effects of diminished immunity typically become apparent in such individuals, who are more frequently kids or teenagers [4].

In theory, some people with type 1 DM may have healthy pancreatic beta cells. But the autoimmune process suppresses them, which is the issue. An inflammatory process in the pancreas causes the initial insult. It happens as a result of immune system cells (T-lymphocytes) invading the islets of Langerhans. T-lymphocytes may identify outsiders in beta cells, which are the bearers of infection, because of a dysfunctional coding process. T-lymphocytes target such cells for destruction, which include beta cells [5]. Transplants of the pancreas, islets of Langerhans, and individual beta cells are all effective treatments for DM. DM can be treated by the use of stem cells. [6].

The immune system's malfunction is the primary contributor to DM. The basis of stem cell therapy for DM is the replacement of beta cells, or dead pancreatic cells, with stem cells. The stem cells become active cells after adhering to the tissues of the pancreas. In DM, a catheter is inserted through the pancreatic artery using an X-ray scanner, which takes 90 minutes [7]. But in the initial stage, a thin needle is used to extract bone marrow from a diabetic's pelvic bone (under local anaesthesia). Stem cells are removed in the lab from the bone marrow. The collected cells are counted, and their quality is evaluated in a lab setting [8]. After commencing treatment, the patient can experience its effects two to three months later. The treatment must be administered once, twice, or more repeatedly for an effective result [9].

The effects of DM stem cell therapy are as follows: 1. It is an expensive procedure and not on the list of requirements for health insurance, according to insurance firms [10]. 2. Recurrent illnesses are brought on by infectious and viral agents against which the body is defenceless [11]. 3. Uncontrolled cell division could lead to oncological diseases [12]. 4. The procedure lacks complete efficacy and evidence and is highly debatable. It is still being developed and will take a lot of time to study and use [13]. The basis of stem cell therapy for DM is the replacement of beta cells, or dead pancreatic cells, with stem cells. The stem cells become active cells after adhering to the tissues of the pancreas [7].

## Review

Treatment of DM with bioresonance therapy

Resonance has been used for many years to treat several diseases [14-18]. Bioresonance therapy is used for disease detection and treatment and was developed by German researchers R. Foll, F. Werner, and H. W. Shimmel [18,19]. Wave copies of various illnesses and organopreparations, which are wave duplicates of healthy organs, are used. In contrast to other works, where we exclusively employed low potencies of nosodes and organopreparations [18,20-22], we used both high and low potencies of nosodes and organopreparations in our research [5-11].

Using nosodes allows for destruction resonance. In the beginning, the patient is tested (diagnosed). A disease nosode, such as stomach cancer, is set on the bioresonance therapy device and tested in a patient [23]. The arrow on display doesn't reach 100 when the nosode is being tested; instead, it drops, for instance, at a value of 40-50. This shows that the patient has been given a stomach cancer diagnosis [24]. Choosing the strength of the nosode stomach cancer in the computer-connected device selector so that it will resonate with the nosode that is tested in the patient's device is the doctor's responsibility when using the bioresonance therapy method. The nosode should match the one for which the patient initially tested positive if it is chosen correctly [25]. The initial nosode, which is in the computer programme of the device for bioresonance therapy, is no longer tested when the stomach cancer nosode is tested in a patient along with the chosen potency of this nosode. This demonstrates the potency of the chosen nosode [23].

Different diseases and organs oscillate at various frequencies. Because it vibrates at the same frequency as the chosen nosode, resonance will result and, for example, will induce the tumour to disappear. In case the chosen nosode's potency is insufficient, first nosode in the actual bioresonance therapy device is currently being investigated (the frequency of oscillations of the nosode is lower than the frequency of oscillations of the tumour). To put it another way, the strategy employed in this case does not result in resonance or elimination of the tumour. The destruction resonance method cannot be used to restore an organ in senile diseases where an organ or part of an organ is destroyed due to the degenerative process. Utilizing techniques that can regenerate tissue or restore ruined organs is required. And the resonance of creation is this process. It has been demonstrated that using the resonance of creation to heal degenerative illnesses like Parkinson's, Alzheimer's, multiple sclerosis, and autoimmune diseases is incredibly beneficial [14-19]. Organo preparation of one or more organs, or a portion of them, can induce a resonance of creation in a patient. With the nosode of this or that disease, it is impossible to produce the resonance of creation [23].

Effective treatment for type 1 DM

The doctor uses a computer selector coupled with bioresonance therapy equipment to find an organ preparation of the pancreas tail (organo preparation) and tests it on a patient being treated for type 1 DM with a bioresonance therapy device. If the organ is healthy, the computer display's arrow will hit the value of 100 and remain there throughout the tests. Similarly, when the organo preparation is tested, the arrow does not rise to a value of 100 if the organ does not function normally; instead, it falls. The surgeon must return the tail of the pancreas, the organ being studied, to its normal state of operation. A resonance between the organo preparation in our computer's selector tested on the patient, and the corresponding organo preparation is required. The matching organo preparation must achieve resonance with the tested organo preparation in the patient. according to the published article and the monographs [6-12]. In order to enter resonance with the organo preparation being tested in the patient, the organo preparation selected for recording from the device selector is typically high potency.

The tail of the pancreas was chosen because of its potency, and as a result, it resonates with the organo preparation being tested in the patient [26]. What does the resonance we produce mean? The new resonance of organopreparations that we have developed does not result in the destruction of the organ, in contrast to the resonance of destruction [24]. It results in the repair of the organ, including the beta cells in the pancreatic tail [27]. The doctor records the information that is available about the organo preparation they have chosen with the needed potency on sugar grains, which are subsequently turned into medicine for the patient. When the patient eats sugar grains, the sick organ is restored. Proof of this can be found in the testing of the research organ throughout the entire course of therapy. When the computer arrow, during testing, gradually approaches the value of 100 without dipping, the doctor realizes the damaged organ is being repaired. Insulin is no longer required when blood sugar levels return to normal simultaneously. The tail of the pancreas was restored after it had fallen into a state of degeneration or pathology thanks to the application of the resonance of creation, and disorders like Parkinson's disease and others associated with senility can also be cured. We knew that hormone replacement therapy could not treat DM until the beta cells in the pancreas that create insulin were repaired [28].

The diabetologist is aware that until the autoimmune process, which is linked to lymphocyte degeneration and dementia, is resolved, a stable cure for DM cannot be achieved. The resonance of creation aids in the treatment of the autoimmune process [14-19]. Because of this, the treatment of type 1 DM and other autoimmune illnesses currently has a good long-term outlook for success when the method of restoring lymphocyte function has been discovered and tested [29].

**Figure 1:**
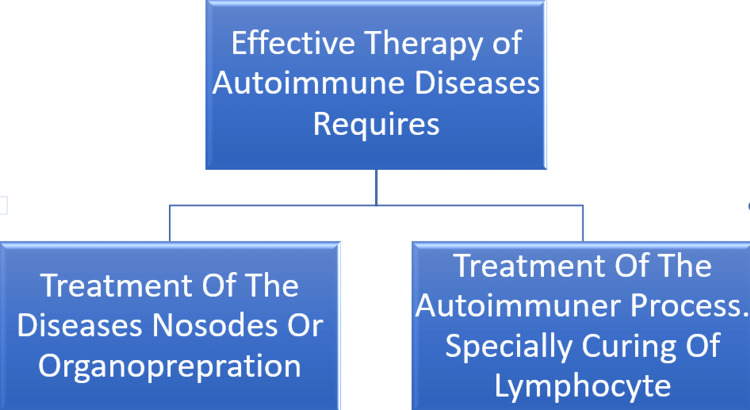
Effective therapy of autoimmune diseases

Therefore, efficient therapy of autoimmune diseases requires at least two components: 1) treatment of the disease's nosodes or organopreparations, and 2) treatment of the autoimmune process, specifically the curing of lymphocytes [23]. Treatment goals are blood sugar stabilization and removal of the need for insulin [30]. Resonance therapy is risk-free and has no negative side effects [31]. Figure 1 shows efficient therapy of autoimmune diseases.

Effective treatment for type 2 DM

In addition to the nosode type 2 DM which includes organopreparations from the pituitary gland, hypothalamus, and diencephalon that were also present in type 1 DM, the same has been evaluated for type 2 DM as well. It is crucial to remember that type 2 DM can also present with multiple sclerosis-like symptoms, as shown by the testing of the nosodes multiple sclerosis and myelin sheath in the organo preparation. It is obvious that the prescription for the treatment of the patients comprised all tested nosodes and organ preparations, including medications for the treatment of the autoimmune process. The destruction resonance approach must first be used to treat amyloidosis to treat type 2 DM. This is because the pancreatic beta cells in this form of DM have amyloid strands attached to them [32].

Because both patients with type 2 DM and those with type 1 DM have high blood sugar levels, the nosodes multiple sclerosis, myelin sheath, hypophysis, hypothalamus, diencephalon, and tail of the pancreas are also evaluated. The patient's treatment regimen included these nosodes and organopreparations. As a consequence, type 2 DM was successfully treated using the resonance of creation and resonance of destruction techniques as well as the nosode autoimmune process [14-19].

Recovery of pancreatic function

Generally, People with type 1 DM experience whole-organ pancreatic degeneration [33]. Such patients require restoration of the whole pancreas, accomplished through the resonance of creation. In patients with type 2 DM, the pancreas is repaired using similar techniques [34]. The task of rebuilding the pancreatic arteries, the lower pancreatoduodenal artery and the upper pancreatoduodenal artery, using the resonance of creation is equally crucial to the therapy of DM [35].

## Conclusions

This article explores how resonance medicine, namely the resonances of destruction and creation, can be used in treating both types of DM as previously reported. A bioresonance method is cost-effective as compared to other methods. This method can be used more effectively to treat both type 1 and type 2 DM. Autoimmune processes were successfully used to treat type 2 DM. Also, to some extent, DM can be abolished by this bioresonance method. This method is not only effective but also safe. The nosode type 2 DM and organopreparations that are part of therapy plan for patients can be used to treat DM. In type 1 DM, patients experience degeneration and they require restoration of complete pancreas that is accomplished with the help of resonance of creation. Similar technique can be used to repair the pancreas of patients with type 2 DM.
